# Maternal Metabolic Status and Vaginal Microbiome Ecology in Early Pregnancy: Associations with Microbial Stability and Birth Outcomes

**DOI:** 10.3390/microorganisms14102341

**Published:** 2026-10-14

**Authors:** Carlos Daniel Mora-Vargas, Tahiri Mendoza-Hernández, Janet Flores-Villanueva, Miguel Angel Diaz-Zurita, Arturo Flores-Pliego, Oscar Villavicencio-Carrisoza, Ana Jimena Lopez-Diaz, Héctor Jesús Borboa-Olivares, Addy Cecilia Helguera-Repetto, Aurora Espejel-Nuñez

**Affiliations:** 1 Departamento de Inmunobioquímica, Instituto Nacional de Perinatología Isidro Espinosa de los Reyes, Ciudad de México 11000, Mexico; mora.vargas.daniel@hotmail.com (C.D.M.-V.); tahiri.mendoza.05@gmail.com (T.M.-H.); janet97flores@gmail.com (J.F.-V.); mikedizuri@gmail.com (M.A.D.-Z.); arturo.flores@inper.gob.mx (A.F.-P.); 2 Escuela Nacional de Ciencias Biológicas, Instituto Politécnico Nacional, Ciudad de México 11350, Mexico; 3 Departamento de Medicina Traslacional, Instituto Nacional de Perinatología Isidro Espinosa de los Reyes, Ciudad de México 11000, Mexico; oscar.villavicencio@inper.gob.mx; 4 Departamento de Medicina Materno-Fetal, Instituto Nacional de Perinatología Isidro Espinosa de los Reyes, Ciudad de México 11000, Mexico; jimenalzdz@gmail.com; 5 Subdirección de Investigación Clínica, Instituto Nacional de Perinatología Isidro Espinosa de los Reyes, Ciudad de México 11000, Mexico; hector.borboa@inper.gob.mx

**Keywords:** vaginal microbiome, early pregnancy microbiota, microbial stability, maternal BMI, gestational weight gain, birth outcomes

## Abstract

The aim of this study was to characterize the vaginal microbiome during early pregnancy according to maternal metabolic status, including prepregnancy body mass index (BMI) and gestational weight gain (GWG), and to explore its relationship with subsequent birth outcomes. A prospective observational cohort study was conducted with sixty-nine pregnant women recruited during the first trimester of pregnancy. Vaginal samples collected between 11 and 13.6 weeks of gestation were analyzed using long-read 16S-rRNA gene sequencing (Oxford Nanopore Technologies). Microbial composition, alpha diversity (richness, Shannon diversity, and evenness), and community structure were evaluated according to prepregnancy BMI, GWG, and birth outcomes. Vaginal microbial communities were predominantly composed of *Lactobacillus* species across all groups; however, distinct ecological configurations were observed according to maternal metabolic status. Women with normal prepregnancy BMI exhibited a greater representation of *Lactobacillus crispatus*, whereas overweight, obesity, and excessive GWG were characterized by a predominance of *Lactobacillus iners*. Maternal obesity was associated with reduced richness and diversity, a trend toward lower community evenness, and enrichment of anaerobic taxa. Women with adverse birth outcomes, particularly preterm birth, exhibited greater community heterogeneity and a higher contribution of non-*Lactobacillus* anaerobes. Overall, maternal metabolic status was associated with distinct vaginal microbiome configurations during early pregnancy. These findings suggest subtle ecological differences in the predominance of Lactobacillus species, community composition, and microbial diversity that may be relevant to subsequent obstetric outcomes.

## 1. Introduction

The vaginal microbiota is a dynamic ecosystem composed of bacteria, archaea, viruses, and fungi that coexist in a symbiotic relationship with the host [1,2]. This microbial community plays a fundamental role in female reproductive health by providing protection against pathogens, modulating local immune responses, and vaginal homeostasis. Its composition is shaped throughout life by hormonal, genetic, environmental, and lifestyle factors, resulting in substantial interindividual variability [3].

In women of reproductive age, the vaginal microbiota is typically characterized by the predominance of *Lactobacillus* species, particularly *Lactobacillus crispatus*, *L. gasseri*, *L. iners*, and *L. jensenii*. These microorganisms contribute to maintaining a low vaginal pH by producing lactic acid and other antimicrobial compounds, thereby limiting the growth of opportunistic pathogens and supporting mucosal health [4,5,6]. However, increasing evidence suggests that not all *Lactobacillus* species confer equivalent ecological properties. While *L. crispatus* is consistently associated with stable and resilient microbial communities, *L. iners* is frequently detected in transitional microbial states and may coexist with anaerobic taxa linked to adverse reproductive outcomes [7].

Pregnancy induces profound physiological, hormonal, and metabolic adaptations that influence the composition of the vaginal microbiota. Elevated estrogen levels promote glycogen accumulation within the vaginal epithelium, creating favorable conditions for *Lactobacillus* proliferation and contributing to the establishment of communities characterized by low diversity and high temporal stability [5,8]. These adaptations are believed to support maternal-fetal health by reinforcing microbial homeostasis during gestation. Nevertheless, deviations from this ecological pattern have been associated with adverse pregnancy outcomes, including spontaneous abortion, premature rupture of membranes, intra-amniotic infection, and preterm birth (PTB). In particular, studies have reported that women who subsequently experience PTB often exhibit lower abundance of *Lactobacillus* species and greater representation of anaerobic bacteria such as *Gardnerella*, *Atopobium*, *Prevotella*, and *Dialister* [5,9,10,11].

Beyond taxonomic composition alone, ecological characteristics such as microbial diversity, community stability, and species dominance have emerged as important indicators of reproductive health. This perspective is particularly relevant during early pregnancy, a critical developmental window in which placentation, maternal metabolic adaptation, and microbial community assembly occur simultaneously. Understanding how vaginal microbial ecology is established during this period may provide important insights into mechanisms underlying pregnancy health and disease [8,12]. Recent evidence further supports an ecological perspective of the maternal microbiome, recognizing that pregnancy outcomes are influenced by complex interactions among microbial communities, maternal metabolism, immune regulation, and inflammatory pathways rather than by the presence of individual microorganisms alone. In particular, alterations in vaginal microbial composition and diversity have been associated with several pregnancy disorders, including preterm birth, gestational diabetes, hypertensive disorders, and spontaneous abortion, highlighting the importance of investigating microbial community structure within the broader context of maternal physiology and pregnancy health [13].

Among the host-related factors proposed to shape these microbial communities, maternal metabolic status has attracted growing attention. Elevated prepregnancy BMI is associated with an increased risk of adverse obstetric outcomes, including PTB, gestational diabetes, hypertensive disorders, and impaired fetal growth. Obesity is associated with endocrine, metabolic, and inflammatory alterations that may affect the vaginal environment and influence microbial community composition. Emerging evidence suggests that obesity is associated with changes in vaginal microbiota structure, including reduced *Lactobacillus* abundance and increased microbial heterogeneity [14,15]. In contrast, considerably less is known about the influence of GWG, despite its potential to reflect dynamic metabolic adaptations occurring throughout pregnancy.

Although several studies have examined the relationship between maternal obesity and the vaginal microbiota, most have focused on broad measures of microbial composition or dysbiosis. Less attention has been given to the ecological characteristics of the vaginal microbiome, including patterns of *Lactobacillus* species dominance, microbial diversity, and community stability during early pregnancy. Furthermore, the combined influence of prepregnancy BMI and GWG on these microbial configurations remains poorly understood.

Therefore, the aim of this study was to characterize the ecological structure of the vaginal microbiome during early pregnancy according to maternal metabolic status, including prepregnancy BMI and GWG, and to explore how these microbial configurations relate to subsequent birth outcomes.

## 2. Materials and Methods

### 2.1. Study Design and Population

This prospective observational cohort study was conducted at the Instituto Nacional de Perinatología in Mexico City from September 2024 to January 2025. A total of 69 pregnant women between 11 and 13.6 weeks of gestation were included and followed prospectively until delivery. Women with chronic autoimmune disease, recent infectious illness, or antibiotic use within the previous 30 days were excluded. One participant was subsequently excluded because she did not continue prenatal follow-up at our Institute, precluding the assessment of gestational weight gain and pregnancy outcome. Consequently, the final analytical cohort consisted of 68 participants. In addition to biological sample collection for microbiome analysis, maternal clinical, obstetric, and neonatal data were obtained from medical records. These data included maternal comorbidities, GWG, pregnancy complications and neonatal outcomes.

For microbiome analyses, participants were classified into three categories. First, according to pre-pregnancy BMI, participants were categorized as underweight (<18.5 kg/m^2^), normal weight (18.5–24.9 kg/m^2^), overweight (25.0–29.9 kg/m^2^), or obese (≥30.0 kg/m^2^). Second, gestational weight gain (GWG) was classified according to the recommendations of the Institute of Medicine and the American College of Obstetricians and Gynecologists (Table 1) [16]. Finally, participants were categorized according to pregnancy outcome as preterm birth (<37 weeks of gestation) or term birth (≥37 weeks of gestation).

### 2.2. Sample Processing

Vaginal samples were collected at patient inclusion using sterile Dacron swabs, preserved in DNA-Shield buffer (1–3 mL) and stored at 4 °C for up to 24 h after collection and at −80 °C for up to two months before processing. Bacterial DNA extraction was performed using the ZymoBIOMICS™ DNA Microprep Kit (Zimo Research, Cat. D4301; Irvine, CA, USA) with the following protocol modifications: Samples were thawed at room temperature and centrifuged at 3500 rpm for 15 min at room temperature. The supernatant was discarded, and 750 μL of ZymoBIOMICS™ Lysis Solution was added to the pellet, which was then transferred to ZR BashingBead™ Lysis Tubes. Then, the tubes were processed using the MP Biomedicals FastPrep-24 system (MP Biomedicals, Santa Ana, CA, USA) in two cycles at 6.5 m/s for 15 s and three cycles at 4 m/s for 40 s, with a 5 min cooldown between cycles. The manufacturer’s protocol was followed from this point onward.

### 2.3. 16S Sequencing Using Nanopore

16S rRNA gene sequencing was conducted on the MinION Mk1B (Oxford Nanopore Technologies ONT, Oxford, UK) using the 16S Barcoding Kit 1-24 (SQK-16S024; ONT, Oxford, UK) and the “Rapid Amplicon Sequencing-16S Barcoding” protocol with minor modifications. The amplification mix included: 10X Taq Buffer 5 μL, dNTPs (5 mM) 2 μL, MgCl2 (25 mM) 3 μL, DNA Taq Polymerase (5 U/μL) 0.7 μL, 16S-barcode primer 10 μL, 100 ng DNA, Nuclease-free water to a final volume of 50 μL. A total of 100 ng from each library was used. Sequencing was conducted using R9.4.1 flow cells (ONT, Oxford, UK). Nanopore sequencing using a MinION Mk1B device (ONT, Oxford, UK) was carried out on an Alienware Aurora R11 computer (Dell Inc.; Round Rock, TX, USA), equipped with an NVIDIA RTX 2080 SUPER (8 GB) graphics processing unit (GPU) and an Intel Core i7-11800H (16-core) 11th-generation processor, enabling local base-calling with Guppy. Sequencing was initiated using MinKNOW version 21.10.4 with high-accuracy base calling and a quality score (Q score) of ≥8, over 5 to 25 h, depending on the amount of data required.

### 2.4. Taxonomic Identification (OTUs)

After sequencing, FASTQ reads were concatenated per sample, trimmed with Nanofilt, and processed using an in-house pipeline in QIIME 2 https://docs.qiime2.org/2024.10/ (accessed on 15 October 2024). The reference database used was the SILVA 138 release (https://www.arb-silva.de/current-release/QIIME2/2025.7; accessed on 3 November 2025).

### 2.5. Statistical Analysis

Data manipulation, statistical analyses, and visualization were conducted using the tidyverse, ggplot2, plotly, vegan, and RColorBrewer packages in R version 4.4.1 (R Foundation for Statistical Computing, Vienna, Austria). Relative abundance profiles were generated at the species level, and bacterial community composition was visualized using stacked bar plots representing the most abundant taxa across prepregnancy BMI categories, GWG groups, and birth outcomes. To facilitate visualization, low-abundance taxa were grouped into a single “Other” category.

Alpha diversity was evaluated using the Shannon diversity index, Pielou’s evenness, and observed species richness (observed OTUs). Diversity analyses and graphical representations were performed using the vegan, ggplot2, tidyverse, and plotly packages.

To identify differentially abundant bacterial species among clinical groups, species-level relative abundances were compared using the non-parametric Kruskal–Wallis rank-sum test. To account for multiple hypothesis testing, *p*-values were adjusted using the Benjamini–Hochberg false discovery rate (FDR) correction. Species with an FDR-adjusted *p*-value < 0.05 were considered statistically significant.

## 3. Results

A total of 68 vaginal samples collected from pregnant women during the first trimester of pregnancy were included in the analysis. Samples were stratified by prepregnancy BMI category (normal weight, overweight, or obese), GWG (inadequate, adequate, or excessive), and birth outcome for comparisons.

Clinical characteristics of total patients are summarized in Table 2. Because only two participants had an underweight pre-pregnancy BMI, this category was excluded from the microbiome analyses due to the insufficient sample size.

After demultiplexing and quality filtering, a total of 1,750,014 forward reads were retained, averaging 28,226 reads per sample. Taxonomic classification allowed the identification of multiple bacterial species commonly associated with the vaginal microbiome. To facilitate visualization and focus on the most relevant taxa, only the most abundant species were included in the graphical analyses, while the remaining taxa were grouped under the category “Other.”

### 3.1. Vaginal Microbiome Composition According to Prepregnancy BMI

Distribution of clinical variables according to prepregnancy BMI are shown in Table S1 (Supplementary Materials, Table S1).

Relative abundance analysis revealed that the vaginal microbiome across all BMI categories was predominantly composed of Lactobacillus species, although differences in the relative contribution of specific taxa were observed (Figure 1).

Among women with normal prepregnancy weight, the vaginal microbiome was mainly dominated by *Lactobacillus crispatus* (34.2%) and *Lactobacillus iners* (25.7%), followed by *Lactobacillus acidophilus* (10.8%). Other taxa detected at moderate abundances included *Amygdalobacter nucleatus* (11.7%) and *Dialister* sp. (9.3%), while the remaining species were present at proportions below 1%.

In the overweight group, *Lactobacillus iners* was the most abundant species, accounting for 43.7% of the microbial community, followed by *Lactobacillus crispatus* (20.9%). Additional Lactobacillus species, including *Lactobacillus jensenii* (7.4%), *Lactobacillus acidophilus* (5.2%), and *Lactobacillus gasseri* (2.7%) were also detected. Non-Lactobacillus taxa, including *Amygdalobacter nucleatus* (6.8%) and *Dialister* sp. (6.8%), contributed moderately to the microbial composition.

Similarly, in women with obesity, the vaginal microbiome was strongly dominated by *Lactobacillus iners*, which represented 44.4% of the total relative abundance. This was followed by *Lactobacillus crispatus* (24.2%) and *Dialister* sp. (16.0%). Other taxa, such as *Lactobacillus acidophilus* (7.3%) and *Lactobacillus jensenii* (3.5%) were present at lower proportions, while the remaining species accounted individually for less than 1% of the microbial community.

Overall, these findings indicate that although *Lactobacillus*-dominated communities were present across all prepregnancy BMI categories, the relative abundance of individual species varied among groups. In particular, *L. crispatus* was more prominent in women with normal BMI, whereas *L. iners* predominated in overweight and obese participants, with a greater contribution from anaerobic taxa such as *Dialister* sp.

Alpha diversity analyses revealed modest differences among prepregnancy BMI groups (Figure S1). Women with normal prepregnancy BMI exhibited slightly higher Shannon diversity and community evenness than women with obesity. This observation did not reflect a reduction in overall Lactobacillus dominance in the overweight or obese groups; rather, it coincided with a shift in species composition characterized by decreased relative abundance of *L. crispatus* and increased predominance of *L. iners*, while *Lactobacillus* remained the dominant genus across all BMI categories.

### 3.2. Vaginal Microbiome Composition According to Gestational Weight Gain

Distribution of clinical variables according to GWG are shown in Table S2 (Supplementary Materials; Table S2).

Across all groups, the vaginal microbiome was predominantly composed of Lactobacillus species, although differences in the relative abundance of specific taxa were observed (Figure 2).

Samples from women with inadequate GWG displayed a comparatively more heterogeneous microbial composition. Although *Lactobacillus iners* remained the most abundant species (35.5%), other taxa were present at higher proportions compared with the other groups, including *Dialister* sp. (19.3%), *Lactobacillus crispatus* (20.6%), and *Amygdalobacter nucleatus* (9.0%). Additional *Lactobacillus* species such as *Lactobacillus acidophilus* (6.8%) were also detected.

Among women with adequate GWG, the microbial community was mainly dominated by *Lactobacillus iners* (37.5%), followed by *Lactobacillus crispatus* (29.1%). Other Lactobacillus species detected included *Lactobacillus acidophilus* (7.9%) and *Lactobacillus jensenii* (4.2%). Non-*Lactobacillus* taxa such as *Amygdalobacter nucleatus* (7.3%) and *Dialister* sp. (7.3%) were also present at moderate relative abundances, while the remaining species represented less than 2% of the microbial composition.

In the excessive GWG group, the vaginal microbiome showed a stronger dominance of *Lactobacillus iners*, accounting for 48.3% of the microbial community. This was followed by *Lactobacillus crispatus* (24.8%), *Dialister* sp. (8.7%), and *Lactobacillus acidophilus* (6.9%). Other taxa, including *Amygdalobacter nucleatus* (3.2%) and *Lactobacillus jensenii* (3.1%), were present at lower relative abundances.

Overall, these findings indicate that *Lactobacillus*-dominated communities were present across all GWG categories, with *Lactobacillus iners* representing the most abundant species. However, women with inadequate gestational weight gain exhibited a higher relative abundance of anaerobic taxa, particularly *Dialister* sp., suggesting increased microbial heterogeneity in this group.

In terms of alpha diversity (Figure S2), women with inadequate GWG displayed a higher number of observed species and greater evenness, reflecting the microbial heterogeneity detected in the relative composition. Conversely, women with excessive weight gain showed reduced diversity, in line with the marked predominance of *Lactobacillus iners*.

### 3.3. Vaginal Microbiome Composition According to Birth Outcome

Distribution of clinical variables according to birth outcome are shown in Table S3 (Supplementary Materials; Table S3).

To investigate whether vaginal microbial composition during the first trimester was associated with birth outcome, samples were stratified into term and preterm birth groups. Overall, the vaginal microbiome in both groups remained predominantly composed of Lactobacillus species, although differences in the relative abundance of specific taxa were observed (Figure 3).

Among women who experienced term birth, the microbial community was strongly dominated by *Lactobacillus iners*, which accounted for 41.2% of the total relative abundance. This was followed by *Lactobacillus crispatus* (23.9%), *Dialister* sp. (11.4%), and *Lactobacillus acidophilus* (6.8%). Additional Lactobacillus species, including *Lactobacillus jensenii* (5.1%), were present at lower proportions, while other taxa represented less than 2% of the microbial community.

In contrast, the vaginal microbiome of women who experienced preterm birth showed a more balanced distribution between the dominant taxa. In this group, *Lactobacillus crispatus* (26.1%) and *Lactobacillus iners* (25.9%) were the most abundant species. Notably, higher relative abundances of non-Lactobacillus taxa were observed, including *Amygdalobacter nucleatus* (15.8%) and *Dialister* sp. (10.0%). Other Lactobacillus species such as *Lactobacillus acidophilus* (8.0%) and *Lactobacillus jensenii* (7.5%) were also present.

Overall, while Lactobacillus-dominated communities were present in both groups, women who experienced preterm birth exhibited a greater contribution of non-Lactobacillus taxa, particularly *Amygdalobacter nucleatus*, and a more evenly distributed microbial composition compared with the strong *L. iners* dominance observed in the term birth group.

The alpha diversity analysis (Figure S3) revealed that women who experienced preterm birth had a higher number of observed species and greater Shannon diversity, suggesting a more diverse and balanced community. This corresponds with the more even distribution between *Lactobacillus crispatus*, *Lactobacillus iners*, and other taxa. In contrast, women with term birth showed lower diversity, consistent with the strong dominance of *Lactobacillus iners*.

Species-level differential abundance analysis using the Kruskal–Wallis test with Benjamini–Hochberg FDR correction, identified *Peptoniphilus indolicus* as the only taxon significantly associated with birth outcome (FDR-adjusted *p* = 0.0396). This species was enriched in the preterm birth group, whereas no other bacterial taxa remained significant after correction for multiple comparisons.

## 4. Discussion

Understanding the role of the vaginal microbiome in early pregnancy has emerged as a critical area of research, particularly in the context of maternal metabolic status and adverse obstetric outcomes. In this study, we characterized the vaginal microbiome during early pregnancy and observed distinct microbial patterns associated with BMI, GWG, and subsequent birth outcomes. Rather than observing marked dysbiosis, our findings revealed subtle yet biologically meaningful differences in microbial community composition, underscoring the importance of moving beyond a simple *Lactobacillus*-versus-non-*Lactobacillus* framework for analyzing the vaginal microbiome during pregnancy and emphasizing the need to consider the predominance of specific *Lactobacillus* species together with the presence of anaerobic taxa.

Several host, lifestyle, and environmental factors have been reported to influence vaginal microbiome composition, including vaginal infections, diet, sexual behavior, smoking status, probiotic use, ethnicity, and physical activity [13,17]. In our study, vaginal infections and smoking status did not differ significantly between the study groups. Although a higher frequency of previous preterm birth was observed among women who subsequently delivered preterm, this finding should be interpreted with caution because only 2 of the 10 preterm birth cases had a history of previous preterm delivery. Consequently, the small number of events precludes drawing firm conclusions regarding this association. Furthermore, because other relevant variables were not systematically assessed, residual confounding cannot be excluded. Future studies involving larger, more diverse cohorts and a more comprehensive characterization of potential confounders are needed to validate these findings.

A key observation was the differential distribution of *Lactobacillus* species across groups. While vaginal communities remained broadly dominated by *Lactobacillus*, individuals with normal BMI were more frequently characterized by *Lactobacillus crispatus*, whereas overweight, obese, and excessive GWG groups showed a relative predominance of *Lactobacillus iners*. This distinction is biologically relevant, as accumulating evidence indicates that not all *Lactobacillus* species confer equivalent ecological or protective functions [7,18]. *L. crispatus* is considered one of the most beneficial constituents of the vaginal microbiome and has consistently been associated with maintenance of vaginal acidity, epithelial homeostasis, and reduced susceptibility to pathogen colonization [7,19,20]. Conversely, *L. iners* has been frequently reported in microbial community configurations associated with greater compositional variability and increased representation of anaerobic bacteria, although its biological role remains incompletely understood [21]. Longitudinal studies have demonstrated that vaginal microbiota dominated by *L. iners* are more prone to instability and conversion to non-*Lactobacillus*-dominated states, whereas *L. crispatus*-dominated communities exhibit greater resilience over time [7].

Although we did not perform a longitudinal analysis, our results raise the possibility that the *L. crispatus*- and *L. iners*-dominated communities observed in our cohort represent distinct ecological states, as previously described in longitudinal studies of the vaginal microbiome. However, this hypothesis requires confirmation through prospective longitudinal analyses.

In fact, the association between *L. iners* and obesity was previously described for reproductive age women [22], reinforcing our findings to look over the pre-pregnancy metabolic state to prevent birth adverse outcomes.

Although obesity has frequently been associated with reduced overall *Lactobacillus* dominance, our findings indicate that this relationship may be more complex during early pregnancy. In our cohort, *Lactobacillus* remained the dominant genus across all BMI categories; the principal difference was observed at the species level, with a change from *L. crispatus* toward *L. iners* in women with overweight and obesity. This finding highlights the importance of examining species-level composition rather than considering *Lactobacillus* as a homogeneous taxonomic group, as individual species differ substantially in their ecological characteristics and their associations with reproductive health.

Incorporating diversity analyses, this study indicates that maternal obesity is associated with a vaginal microbial profile characterized by reduced richness and diversity, a trend toward lower community evenness, predominance of *L. iners*, and enrichment of anaerobic taxa, particularly members of the genus *Dialister*. Collectively, these microbial characteristics resemble community configurations that have been associated in longitudinal studies with reduced ecological stability of the vaginal microbiome. However, given the cross-sectional design of the present study, microbial stability could not be directly assessed, and these observations should be interpreted as ecological associations rather than evidence of temporal microbial dynamics.

One of the most interesting findings within this anaerobic component was the increased representation of *Dialister*. Although *Dialister* is not considered a primary vaginal pathogen, it has consistently been detected as a member of anaerobic polymicrobial communities associated with vaginal dysbiosis and adverse reproductive outcomes [23]. Previous studies have reported an increased abundance of *Dialister* in obese women together with reduced *Lactobacillus* predominance, suggesting that this genus reflects broader ecological alterations of the vaginal microbiome associated with metabolic dysfunction rather than acting as an isolated pathogenic taxon [24]. Moreover, *Dialister* species have been identified among the bacterial taxa enriched in women who subsequently develop preterm birth, further supporting their potential role as components of microbial community configurations associated with adverse pregnancy outcomes [11]. Accordingly, the enrichment of *Dialister* observed in our overweight and obese participants may represent an ecological signature of altered vaginal microbial community composition linked to maternal metabolic status rather than a species-specific effect.

GWG also appeared to contribute to these microbial configurations in our cohort. Notably, excessive GWG was associated with microbial profiles characterized by *L. iners*-dominance, while inadequate GWG was linked to increased inter-individual variability and community heterogeneity.

The association between GWG and the vaginal microbiome is still poorly understood, and available data remain limited. However, emerging evidence suggests that GWG may influence microbiota composition independently of pre-pregnancy BMI, reflecting dynamic metabolic adaptations during pregnancy [25]. In line with these observations, the current results support the hypothesis that maternal nutritional and metabolic adaptation can influence the reproductive tract microbiome from the earliest stages of gestation [24,26]. In this context, our results further suggest that early pregnancy may represent a critical period during which maternal metabolic factors are associated with distinct vaginal microbial configurations that might interact to shape the trajectory of the vaginal ecosystem. These interactions may, in turn, influence susceptibility to adverse outcomes such as preterm birth (PTB).

In our cohort, increased community heterogeneity and the presence of anaerobic taxa were more frequently observed in individuals with adverse metabolic profiles and in those associated with PTB. While the role of microbial diversity in the vaginal microbiome is complex, higher diversity has frequently been associated with microbial community configurations considered less stable in longitudinal studies of the vaginal microbiome [12,27]. Multiple studies have reported that increased vaginal microbial diversity during early pregnancy is associated with a higher risk of PTB and other adverse outcomes [8,28]. Similarly, meta-analyses and cohort studies have demonstrated that both diverse, non-*Lactobacillus*-dominated communities and *L. iners*-dominated profiles are associated with increased PTB risk when compared to *L. crispatus*-dominated states [29,30].

Interestingly, species-level differential abundance analysis identified *Peptoniphilus indolicus* as the only taxon significantly enriched in the PTB group. *Peptoniphilus* species are anaerobic Gram-positive bacteria detected in dysbiotic vaginal communities and associated with inflammatory conditions of the female reproductive tract [11], as well as with a variety of opportunistic infections [31]. Evidence regarding the role of this microorganism during pregnancy remains limited. Nevertheless, a case report described a woman at 22 weeks of gestation who presented with fever, chills, nausea, and abdominal pain, rapidly progressing to spontaneous abortion. Histopathological examination of the placenta demonstrated acute chorioamnionitis, suggesting a potential association between this microorganism and inflammatory processes implicated in adverse pregnancy outcomes, including preterm birth [32]. However, given the limited number of preterm birth cases in our cohort and the exploratory nature of this finding, its biological significance should be interpreted with caution and warrants further investigation in larger longitudinal studies.

An additional finding deserving further consideration was the predominance of *Lactobacillus iners* not only among women with adequate gestational weight gain but also among those who subsequently experienced term birth. Although *L. iners* has frequently been associated with transitional vaginal microbial communities and an increased risk of adverse pregnancy outcomes, this species is also commonly detected in healthy reproductive-age women and uncomplicated pregnancies, particularly when considered within the broader microbial community structure [33]. In our cohort, the predominance of *L. iners* among women with favorable pregnancy outcomes indicates that its presence alone should not be considered a marker of vaginal dysbiosis. This finding is consistent with our previous study in healthy Mexican pregnant adolescents, in which *L. iners* was also the predominant *Lactobacillus* species within the urogenital microbiome [34]. Collectively, these observations support the growing evidence that the biological significance of *L. iners* is context dependent and cannot be inferred from its presence alone. Rather, its role appears to be determined by the surrounding microbial community, host metabolic and immunological factors, and population-specific characteristics. These findings reinforce the importance of interpreting species-level microbial patterns within an ecological framework rather than attributing a uniformly beneficial or detrimental role to individual taxa.

Overall, our findings do not suggest a direct causal relationship between specific bacterial taxa and adverse pregnancy outcomes but rather support an ecological interpretation of the observed microbial community composition. Specifically, the predominance of *L. iners* together with the increased representation of anaerobic taxa in some clinical groups is consistent with microbial configurations that have previously been associated with increased PTB risk in longitudinal studies [35]. Accordingly, the coexistence of *Lactobacillus* species with anaerobic bacteria observed in our cohort may represent ecological configurations resembling those reported in women at greater risk of vaginal dysbiosis and adverse pregnancy outcomes. Because our study was based on a single microbiome assessment during early pregnancy, these observations should be interpreted as associations rather than evidence of temporal microbial dynamics or causal ecological transitions.

From a clinical perspective, our findings highlight the potential of the vaginal microbiome as an early biomarker of pregnancy risk. The identification of specific ecological patterns, such as reduced *L. crispatus* prevalence, increased *L. iners* dominance, and higher community heterogeneity, may provide valuable insights into maternal health and pregnancy outcomes. However, further longitudinal studies are needed to determine whether these microbial signatures have predictive value and can be targeted by therapeutic interventions.

Another important consideration is that the associations observed in this study likely reflect complex, multifactorial processes. The vaginal microbiome is influenced by a wide range of factors, including hormonal changes, immune modulation, sexual behavior, and environmental exposures [36]. Moreover, pregnancy itself induces profound physiological adaptations that can influence microbial community composition throughout gestation [12]. An important consideration when interpreting our findings is the cross-sectional nature of the microbiome assessment. Vaginal samples were collected at a single time point during the first trimester; therefore, temporal changes in microbial composition, ecological stability, or transitions between community configurations could not be directly evaluated. Consequently, our findings should be interpreted as associations between early pregnancy microbial configurations and maternal metabolic status rather than evidence of causal or longitudinal microbial processes. Future studies incorporating repeated sampling throughout pregnancy will be essential to determine whether the microbial configurations identified here remain consistent over time and to clarify their relationship with pregnancy outcomes.

## 5. Conclusions

Our findings support the idea that the vaginal microbiome during early pregnancy reflects, at least in part, the maternal metabolic environment. Rather than identifying a clearly dysbiotic state, we observed distinct microbial community configurations characterized by differences in *Lactobacillus* dominance, community composition, and the relative abundance of anaerobic taxa. These findings are consistent with recent reviews proposing that pregnancy disorders arise from complex interactions between host physiology and microbial ecology rather than from the presence of individual pathogenic taxa.

Together, our findings demonstrate that maternal metabolic status is associated with distinct vaginal microbial configurations during early pregnancy. Although the cross-sectional design precludes assessment of temporal microbial dynamics or causal relationships, these observations contribute to a growing body of evidence supporting an ecological perspective of the vaginal microbiome in pregnancy. Future longitudinal studies incorporating repeated sampling throughout gestation will be essential to determine how these microbial configurations evolve over time and whether they have predictive value for adverse obstetric outcomes.

## 6. Limitations

This study has some limitations that should be acknowledged. First, the use of 16S rRNA gene sequencing does not provide information on microbial functional activity or strain-level variation. Second, because the vaginal microbiome was assessed at a single time point during the first trimester, temporal changes in microbial composition, ecological stability, and community resilience could not be directly evaluated. Additionally, the limited subgroup sizes may have reduced statistical power and increased the risk of both type I and type II errors. Therefore, our findings should be considered hypothesis-generating and interpreted with caution until they are validated in larger, preferably multicenter, prospective cohorts.

## Figures and Tables

**Figure 1:**
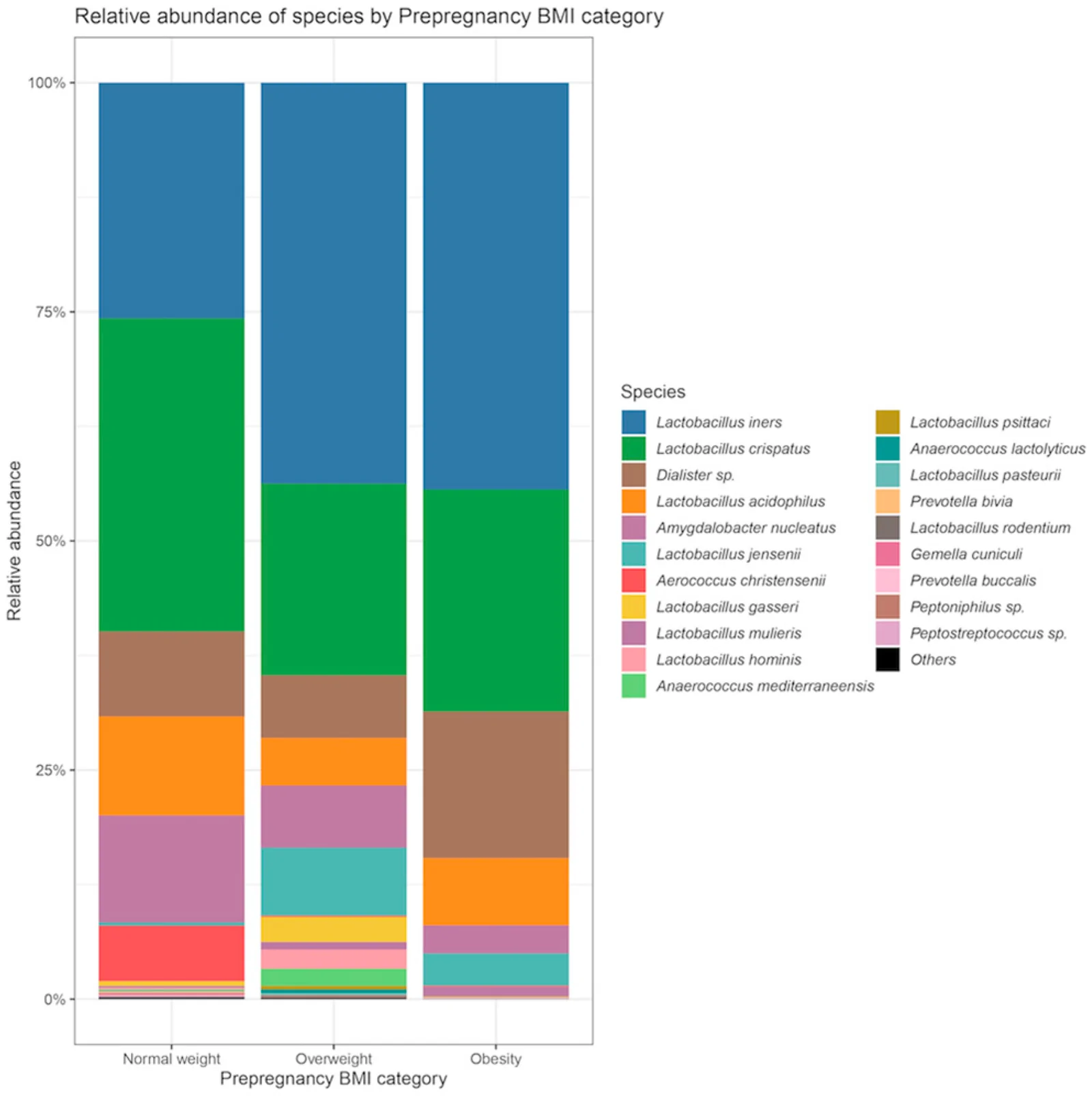
Vaginal microbiome profile during the first trimester according to prepregnancy BMI category, based on 16S rRNA gene sequencing. Stacked bar plots display the sequence abundances of the most abundant bacterial species across the different prepregnancy BMI categories (normal weight, overweight, and obesity). The y-axis represents the relative abundance expressed as a percentage for each bacterial taxon, while the x-axis shows the BMI categories analyzed in the study. To facilitate visualization, only the most abundant species are displayed, while the remaining taxa are grouped under the category “Other.”.

**Figure 2:**
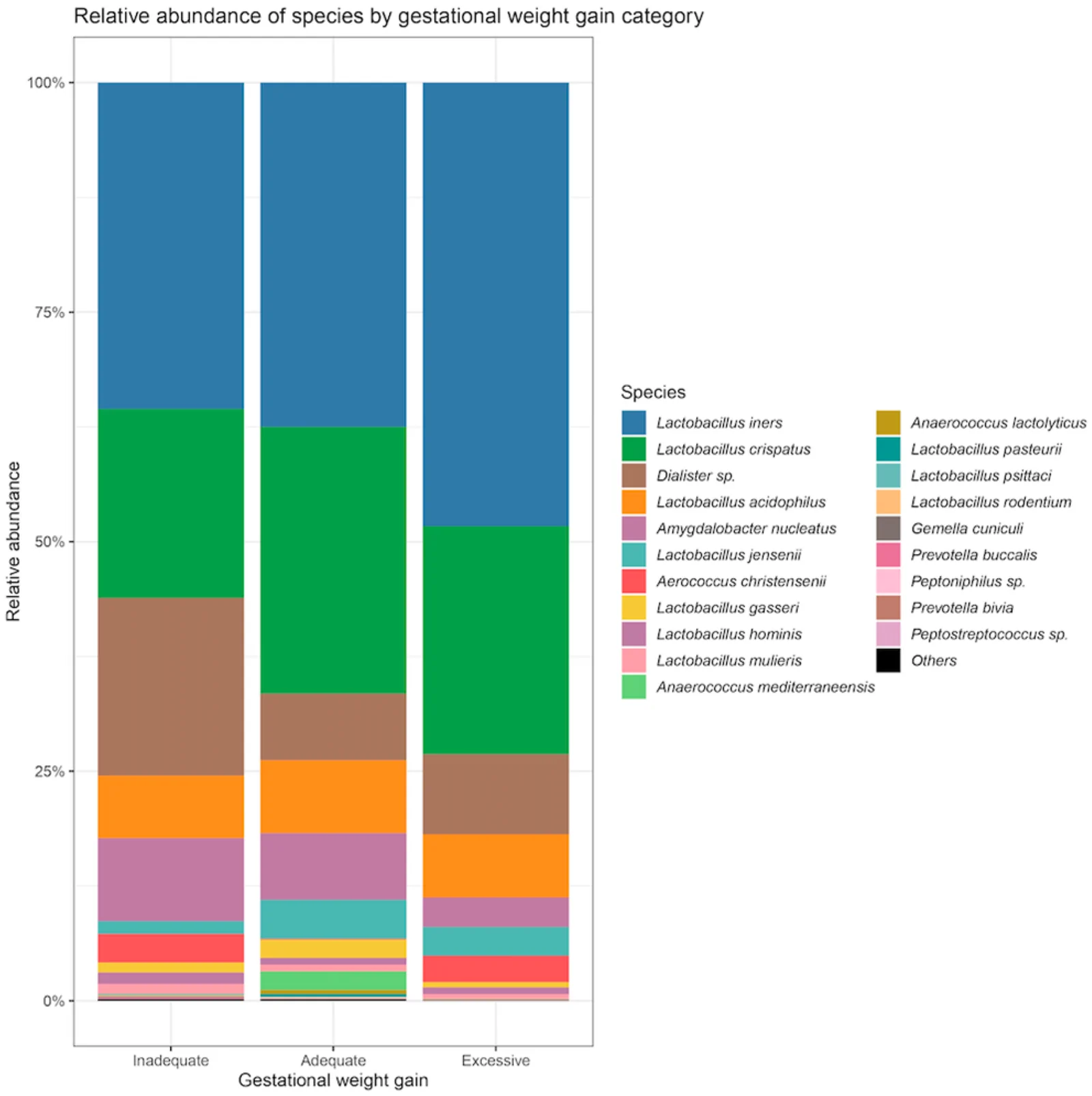
Vaginal microbiome profile during the first trimester according to gestational weight gain, based on 16S rRNA gene sequencing. Stacked bar plots show the relative abundances of the most abundant bacterial species across the three gestational weight gain categories (inadequate, adequate, and excessive). The y-axis represents the relative abundance expressed as a percentage for each bacterial taxon, while the x-axis shows the gestational weight gain categories included in the study. To facilitate visualization and focus on the most relevant taxa, only the most abundant species are displayed, while the remaining taxa are grouped under the category “Other.”.

**Figure 3:**
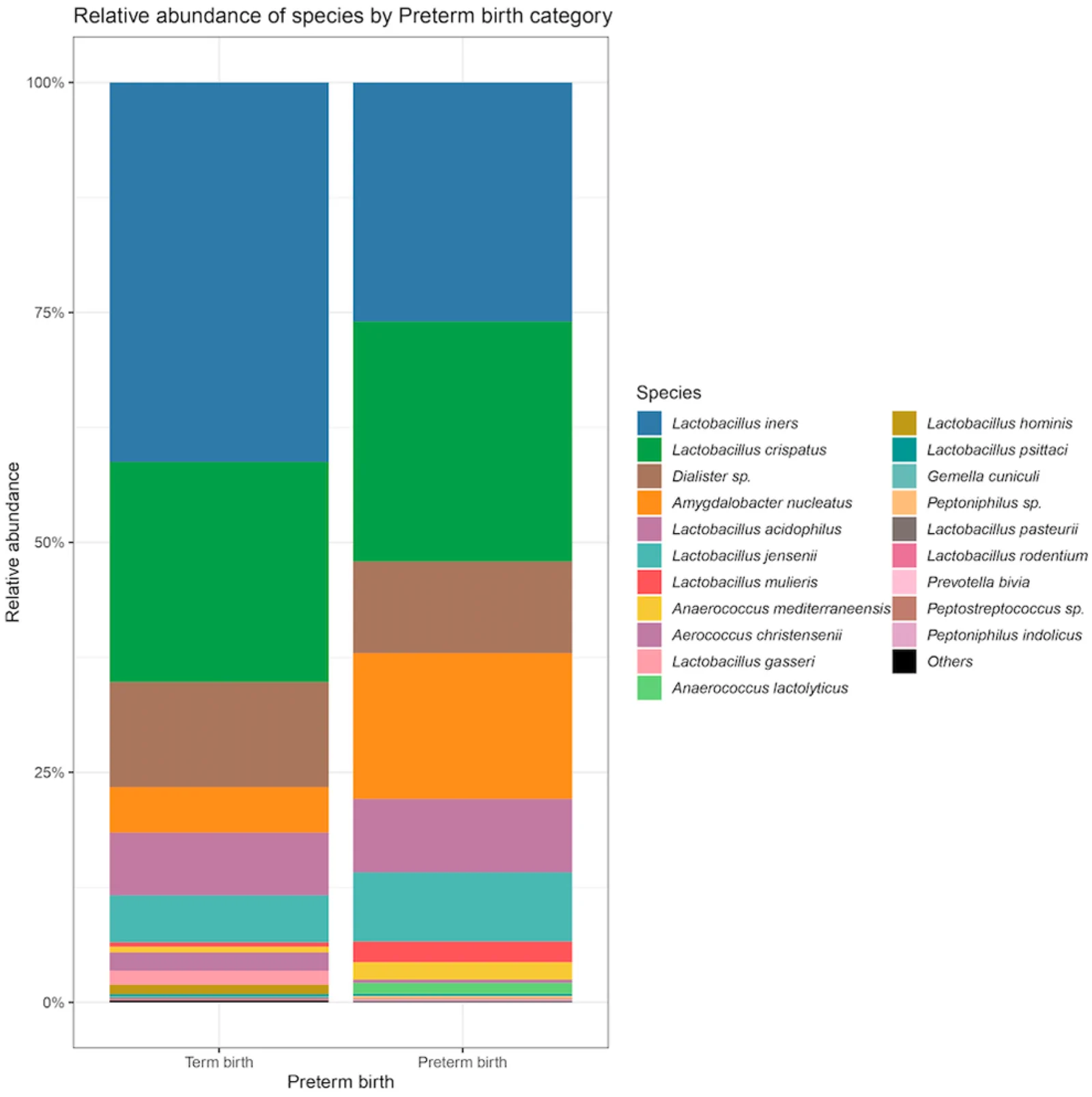
Vaginal microbiome profile during the first trimester according to birth outcome, based on 16S rRNA gene sequencing. Stacked bar plots show the relative abundances of the most abundant bacterial species in women with term and preterm birth. The y-axis represents the relative abundance expressed as a percentage for each bacterial taxon, while the x-axis shows the birth outcome categories included in the study. To facilitate visualization and focus on the most relevant taxa, only the most abundant species are displayed, while the remaining taxa are grouped under the category “Other.”.

**Table 1:** Recommended Pregnancy Weight Gain According to Pregestational BMI.

Prepregnancy Weight Category	Prepregnancy Body Mass Index (kg/m^2^)	Recommended Range of Total Weight (lb)	Recommended Rates of Weight Gain in the Second and Third Trimester (lb) (Mean Range [lb/wk])
Underweight	Less than 18.5	28–40	1 (1–1.3)
Normal Weight	18.5–24.9	25–35	1 (0.8–1)
Overweight	25–29.9	15–25	0.6 (0.5–0.7)
Obese	30 and greater	11–20	0.5 (0.4–0.6)

Calculations assume a 1.1–4.4 lb weight gain in the first trimester [16].

**Table 2:** Descriptive characteristics of the study population.

Variable	Category	Population: N = 68. Median (IQR) or n (%)
Maternal age (years)		30.5 (26.0–35.0)
Prepregnancy BMI	Underweight	2 (2.9%)
	Normal	20 (29.4%)
	Overweight	28 (41.2%)
	Obesity	18 (26.5%)
Gestational Weight Gain	Inadequate	26 (38.2%)
	Adequate	25 (36.8%)
	Excessive	17 (25.0%)
Cesarean Section		46 (67.6%)
Preeclampsia		9 (13.2%)
Gestational Diabetes		13 (19.1%)
HIV Infection		2 (2.9%)
Ureaplasma Vaginitis		38 (55.9%)
Mycoplasma Vaginitis		3 (4.4%)
Candidiasis		20 (29.4%)
Bacterial Vaginosis		10 (14.7%)
Tobacco Use		5 (7.4%)
Exercise During Pregnancy		24 (35.3%)
Preterm Birth		10 (14.7%)
History of Preterm Birth		3 (4.4%)
History of Preeclampsia		5 (7.4%)
History of Gestational Diabetes		5 (7.4%)

## Data Availability

The datasets used and/or analyzed during the current study are available from the corresponding author on reasonable request.

## Supplementary Materials

The following supporting information can be downloaded at: https://www.mdpi.com/article/10.3390/microorganisms14102341/s1, Figure S1: Alpha diversity of the first-trimester vaginal microbiota across prepregnancy body mass index categories. Figure S2: Alpha diversity of the first-trimester vaginal microbiota across gestational weight gain categories. Figure S3: Alpha diversity of the first-trimester vaginal microbiota according to term or preterm birth outcome. Table S1: Clinical characteristics of participants according to pre-pregnancy BMI. Table S2: Clinical characteristics of participants according to gestational weight gain (GWG). Table S3: Clinical characteristics of participants according to pregnancy outcome (Preterm birth).

## Abbreviations

The following abbreviations are used in this manuscript:

BMI	Body mass index
GWG	Gestational weight gain
PTB	Preterm birth
