# Supplementary material for: Maternal Metabolic Status and Vaginal Microbiome Ecology in Early Pregnancy: Associations with Microbial Stability and Birth Outcomes
Source: Microorganisms. 2026 Oct 14;14(10):2341. doi: 10.3390/microorganisms14102341 (PMC13707807; doi:10.3390/microorganisms14102341)
Supplement: Supplementary file 1 [file microorganisms-14-02341-s001.zip › SUPPLEMENTARY MATERIAL.pdf]

SUPPLEMENTARY MATERIAL

Supplementary Table S1. Clinical characteristics of participants according to pre-pregnancy BMI.

		**Body Mas Index Classification**			
**Characteristics**	**Category**	**Low Weight, n = 2 n (%)**	**Normal Weight, n = 20 n (%)**	**Overweight, n = 28 n (%)**	**Obesity, n = 18 n (%)**
Gestational Weight Gain	Inadequate	2 (100.0%)	12 (60.0%)	8 (28.6%)	4 (22.2%)
	Adequate	0 (0.0%)	7 (35.0%)	14 (50.0%)	4 (22.2%)
	Excessive	0 (0.0%)	1 (5.0%)	6 (21.4%)	10 (55.6%)
Cesarean Section		1 (50%)	10 (50.0%)	20 (71.4%)	15 (83.3%)
Preeclampsia		0 (0.0%)	0 (0.0%)	3 (10.7%)	6 (33.3%)
Gestational Diabetes		0 (0.0%)	3 (15.0%)	7 (25.0%)	3 (16.7%)
HIV Infection		1 (50.0%)	1 (5.0%)	0 (0.0%)	0 (0.0%)
Ureaplasma Vaginitis		1 (50.0%)	12 (60.0%)	14 (50.0%)	11 (61.1%)
Mycoplasma Vaginitis		0 (0.0%)	0 (0.0%)	1 (3.6%)	2 (11.1%)
Candidiasis		1 (50.0%)	3 (15.0%)	7 (25.0%)	9 (50.0%)
Bacterial Vaginosis		1 (50.0%)	4 (20.0%)	3 (10.7%)	2 (11.1%)
Tobacco Use		0 (0.0%)	1 (5.0%)	0 (0.0%)	4 (22.2%)
Exercise During Pregnancy		0 (0.0%)	6 (30.0%)	10 (35.7%)	8 (44.4%)
Preterm Birth		1 (50%)	2 (10%)	3 (10.7%)	4 (22.2%)
History of Preterm Birth		0 (0.0%)	0 (0.0%)	2 (7.1%)	1 (5.6%)
History of Preeclampsia		0 (0.0%)	1 (5.0%)	2 (7.1%)	2 (11.1%)
History of Gestational Diabetes		0 (0.0%)	0 (0.0%)	3 (10.7%)	2 (11.1%)

Supplementary Table S2. Clinical characteristics of participants according to gestational weight gain (GWG).

		Maternal Gestational Weight Gain, n = 68		
**Characteristics**	**Category**	**Inadequate, n = 26 n (%)**	**Adequate, n = 25 n (%)**	**Excessive, n = 17 n (%)**
Prepregnancy BMI	Low	2 (7.7%)	0 (0%)	0 (0%)
	Normal	12 (46.2%)	7 (28%)	1 (5.9%)
	Overweight	8 (30.8%)	14 (56%)	6 (35.3%)
	Obesity	4 (15.4%)	4 (16%)	10 (58.8%)
Cesarean Section		16 (61.5%)	17 (68%)	13 (76.5%)
Preeclampsia		3 (11.5%)	0 (0%)	6 (35.3%)
Gestational Diabetes		9 (34.6%)	0 (0%)	4 (23.5%)
HIV Infection		2 (7.7%)	0 (0%)	0 (0%)
Ureaplasma Vaginitis		16 (61.5%)	11 (44%)	11 (64.7%)
Mycoplasma Vaginitis		2 (7.7%)	0 (0%)	1 (5.9%)
Candidiasis		11 (42.3%)	5 (20%)	4 (23.5%)
Bacterial Vaginosis		6 (23.1%)	3 (12%)	1 (5.9%)
Tobacco Use		2 (7.7%)	0 (0%)	3 (17.6%)
Exercise During Pregnancy		9 (34.6%)	7 (28%)	8 (47.1%)
Preterm Birth		4 (15.4%)	3 (12%)	3 (17.6%)
History of Preterm Birth		1 (3.8%)	1 (4%)	1 (5.9%)
History of Preeclampsia		1 (3.8%)	2 (8%)	2 (11.8%)
History of Gestational Diabetes		2 (7.7%)	0 (0%)	3 (17.6%)

Supplementary Table S3. Clinical characteristics of participants according to pregnancy outcome (Preterm birth).

Characteristics	Category	Preterm birth, n = 10
Prepregnancy BMI	Low	1 (10.0%)
	Normal	2 (20.0%)
	Overweight	3 (30.0%)
	Obesity	4 (40.0%)
Gestational Weight Gain	Inadequate	4 (40.0%)
	Adequate	3 (30.0%)
	Excessive	3 (30.0%)
Preeclampsia		2 (20.0%)
Gestational Diabetes		3 (30.0%)
HIV Infection		0 (0.0%)
Ureaplasma Vaginitis		5 (50.0%)
Mycoplasma Vaginitis		0 (0.0%)
Candidiasis		4 (40.0%)
Bacterial Vaginosis		2 (20.0%)
Tobacco Use		1 (10.0%)
Exercise During Pregnancy		5 (50.0%)
History of Preterm Birth*		2 (20.0%)
History of Preeclampsia		0 (0.0%)
History of Gestational Diabetes		1 (10.0%)

* Only two participants in the entire cohort had a history of previous preterm birth, both of whom were in the preterm birth group. Although the difference was statistically significant ($p < 0.05$), the small number of cases precludes drawing robust conclusions.
